# Swallowing, voice and quality of life of patients submitted to extended supratracheal laryngectomy

**DOI:** 10.31744/einstein_journal/2020AO5390

**Published:** 2020-05-13

**Authors:** Guilherme Maia Zica, Andressa Silva de Freitas, Ana Catarina Alves e Silva, Fernando Luiz Dias, Izabella Costa Santos, Emilson Queiroz Freitas, Hilton Augusto Koch

**Affiliations:** 1 Instituto Nacional de Câncer Rio de JaneiroRJ Brazil Instituto Nacional de Câncer , Rio de Janeiro , RJ , Brazil .; 2 Fundação Oswaldo Cruz Rio de JaneiroRJ Brazil Fundação Oswaldo Cruz , Rio de Janeiro , RJ , Brazil .

**Keywords:** Laryngectomy, Treatment outcome, Deglutiton, Voice, Quality of life

## Abstract

**Objective:**

To describe functional and quality of life results after extended supratracheal laryngectomy.

**Methods:**

In the period from September 2009 to January 2018, 11 male subjects were submitted to extended supratracheal laryngectomy. Swallowing abilities were assessed through videofluoroscopy and the clinical scale Functional Communication Measures of Swallowing. The voices were classified by means of the perceptual-auditory analysis Consensus Auditory-Perceptual Evaluation of Voice. All subjects completed a self-assessment questionnaire for voice and swallowing.

**Results:**

Aspiration was found in four patients and all presented stasis in different structures. All subjects in this study were exclusively orally fed and hydrated. In the evaluation of quality of life in swallowing, patients had mean >80 in all areas (83.47 mean of scores). The general degree and the presence of roughness were the highest means present in Consensus Auditory-Perceptual Evaluation of Voice (37.81 and 49.36, respectively). The mean of 33.36 (±22.56) had little impact on quality of life under the perspective of vocal aspects.

**Conclusion:**

After supratracheal laryngectomy, swallowing was sufficiently restored and the quality of life was satisfactory. The voice presents severely impaired quality and preserved oral communication, with low impact on the activities of daily living. All individuals who maintained two cricoarytenoid units presented better functional results in swallowing and voice.

## INTRODUCTION

Laryngeal cancer is the second most common respiratory malignancy in the world, and has one of the highest incidences of head and neck tumors. It is present mostly in men aged over 40 years and its most prevalent histological type is squamous cell carcinoma, confirmed in more than 90% of cases.^( 1 , 2 )^ The prognosis is limited when the disease is diagnosed in the advanced stages, impairing quality of life (QoL), with regard to speech and swallowing disorders.^( 1 , 3 - 6 )^

Treatment of laryngeal carcinoma with total laryngectomy (TL), with or without radiation therapy (RT), significantly changes the patient’s QoL. Complete removal of the organ causes major impacts, on phonation and swallowing abilities.^( 7 , 8 )^ Studies show RT and chemotherapy (CT) as an alternative to primary laryngectomy, thus avoiding disfiguration, permanent tracheostoma and complications, such as pharyngocutaneous fistula, infections and rupture of the carotid artery.^( 6 - 9 )^ However, there is evidence, especially in advanced staging, that the sequelae of these techniques can also be drastic, leading to the preservation of a non-functional organ with significant structural alterations.^( 8 , 10 )^

In view of the failure of RT treatment and low efficacy of non-surgical protocols with high levels of toxicity, open horizontal partial laryngectomies are performed in specialized centers as a viable alternative to the other procedures.^( 10 )^ This conservative surgical technique for laryngeal carcinoma removes the tumor and its safety margin free of the disease, reconstructing a functional neolarynx.^( 3 , 10 , 11 )^

Horizontal partial laryngectomies have been established as surgical options for the treatment of laryngeal cancer in the intermediate/advanced stages.^( 12 )^ In 1959, the supracricoid laryngectomies (SCL) with cricohyoidoepiglottopexy (CHEP) was introduced by Majer, as a safe oncologic resection for patients with laryngeal cancer.^( 13 )^ As a current and analogous method, supratracheal laryngectomies (STL) arose for the treatment of laryngeal tumors with subglottic extension and anterior invasion of cricoid cartilage.^( 3 , 14 , 15 )^

The surgical technique of STL consists of resection of the thyroid cartilage and its paraglottic space, preservation of the posterior part of the cricoid cartilage, maintenance of the hyoid bone, epiglottis and at least one arytenoid cartilage.^( 12 , 13 , 16 )^ Its reconstruction is described with two variations: tracheohyoidopexy (THP), which consists of the maintenance of both or only one cricoarytenoid unit (CAU); tracheohyoidoepiglottopexy (THEP) with preservation of the epiglottis, maintaining two or only one CAU.^( 17 , 18 )^ In cases in which resection is broader than that predicted by the described technique, as in STL reports with extension to the base of tongue, adjacent tissue or arytenoids, we add to the nomenclature the term “extended” or “modified” STL.^( 19 - 21 )^

In the specialized literature, there is evidence of functional complications following horizontal partial laryngectomies, in regarding to breathing, voice and swallowing, with a low incidence of permanent tracheostomies.^( 9 , 22 )^ However, the correlation of functional effects on QoL of patients undergoing STL, to date, has only been suggested in a single study, conducted in 2015.^( 23 )^ Their preliminary findings described the effectiveness of the technique in a group of 22 patients, in Italy, with exclusive maintenance of oral feeding in 20 patients, and better results in voice and QoL compared to swallowing scores due to changes in diet.^( 23 )^ It is therefore of paramount importance that the population of developing countries is described/included with respect to their real conditions and socio-economic and demographic contexts.

## OBJECTIVE

To evaluate and describe the functional and quality of life results of swallowing and voice of patients undergoing extended supratracheal laryngectomy with tracheohyoidepiglottopexy in a Latin American cancer hospital.

## METHODS

This is an observational cross-sectional study, approved by the Research Ethics Committee of the organization under CAAE: 26331314.2.0000.5274, opinion 616.249. Individuals of both sexes, enrolled in the period from 2009 to 2018, were included in the Head and Neck Department of the *Hospital do Câncer I* of the *Instituto Nacional de Câncer* , in the city of Rio de Janeiro (RJ), who underwent STL with extended THEP reconstruction. Exclusion criteria comprised patients aged under 18 years, non-cooperative patients due to linguistic-cognitive impairments, and those who underwent another type of surgical intervention in the laryngeal region after STL with extended THEP. All volunteers participating in the study signed an Informed Consent Form.

Individuals eligible for the study were found in surgical records according to the pre-established timeframe. Demographic and clinical information was collected by reviewing physical and electronic records. For speech-language and QoL evaluations, patients were scheduled and participated in the procedures described below.

### Dynamic and quantitative deglutition analysis

Videofluoroscopic swallowing studies (VFSS) were used to evaluate swallowing of the patients in the study. Studies were performed with the Siemens AXIOM Iconos Remote Control X-ray (serial number 13020) and evaluated with the videofluoroscopic deglutition evaluation protocol, based on Logemann.^( 24 )^ All video segments were recorded in lateral view, with an image capture rate of 30 frames per second.

The preparation of the consistencies was made as follows: contrast was offered in a glass, with free handling and using dilutions of barium sulphate (BS) 100% Bariogel^®^, mineral water and thickener Resource^®^ ThickenUp Clear. We evaluated patients with three consistencies: 5mL liquid (2.5mL water + 2.5mL BS), 10mL (5mL water + 5mL BS) and 20mL (10mL water + 10mL BS); thickened liquid in 5mL of BS, 10mL of BS and 20mL of SB; and puree in 5mL (5mL BS + 1.2g Thickener), 10mL (10mL SB + 2.4g Thickener) and 20mL (20mL SB + 3.6g Thickener). In order to standardize the test, the solid consistency was not included, due to the existence of individuals with missing dentition. The Penetration-Aspiration Scale (PAS), developed by Rosenbek et al., was used to analyze the videofluoroscopic examination.^( 25 )^

The Functional Communication Measures of Swallowing (FCM) from the American Speech Language Hearing Association (ASHA) National Outcomes Measures Scale (NOMS) is based on clinical observation and widely accepted in the evaluation of oropharyngeal dysphagia, and its scores range from 1 (less functional) to 7 (normal). It was used as a subjective and complementary means of oral ingestion analysis.^( 26 )^

The only tool available to evaluate the effects of swallowing changes on subjects undergoing head and neck cancer treatment is the M. D. Anderson Dysphagia Inventory (MDADI) questionnaire. This was developed and validated by Chen et al.,^( 27 )^ and comprises 20 questions − one global, and the others divided as emotional, functional and physical domains.^( 28 , 29 )^ All scales have five response possibilities, ranging from 1 (totally agree) to 5 (totally disagree), with the exception of an emotional subscale, item (E7) and a functional subscale, item (F2), which range from 5 (totally agree) to 1 (totally disagree). The score of each subscale is obtained from the calculation of the mean score of their items, multiplied by 20. The total score is obtained from the mean scores of each domain (emotional, functional and physical) multiplied by 20. The individual can obtain a score ranging from zero to one hundred; in that, the lower the value of the observed score, the worse the effect of dysphagia on their QoL and functionality.^( 27 , 28 )^

### Perceptual evaluation and quality of life in voice

The patients in this study were evaluated for vocal quality using a method of perceptual-auditory analysis developed by Kempster et al.,^( 29 )^ the Consensus Auditory-Perceptual Evaluation of Voice (CAPE V). The aim of this scale is to qualify the sound signal, allowing the vocal quality of the individual to be determined in the context of the study of voice. The listener’s analysis is passed to a 100mm line scale with scores determined by the location of the chosen mark (ranging from zero to one-hundred) by a previously trained speech-language pathologist. The evaluated aspects are represented by the general degree of the patient’s voice, presence and degree of roughness, breathiness and tension, and pitch and loudness variations. The closer to one hundred the value of the score obtained, the worse the vocal quality of the patient.^( 29 )^

To investigate the impact of voice changes on patient’s QoL, the questionnaire *Índice de Desvantagem Vocal* (IDV) was applied. It was translated and adapted into Brazilian Portuguese from the Voice Handicap Index (VHI), by Behlau et al.,^( 30 )^ and allows interpreting the patient’s perception of dysphonia and repercussions on their QoL. The protocol is composed of 30 items that investigate 3 domains − functional (10), organic (10) and emotional (10) −, quantified by a 5-point Likert scale (0-4) and its score is calculated by a simple summation. The results obtained can vary from zero to 120 and, the greater the score, the more important is the impairment in their QoL.^( 30 )^

## RESULTS

A total of 11 patients were evaluated, with a mean age of 67.45 years (±8.5 years) and a median of 69 years, 9 self-reported Caucasian (81.8%) and 2 African descendent (18.2%) ( Table 1 ). Of the group studied, 45.5% confirmed a family history of cancer. The majority of the individuals had histopathological diagnosis of squamous cell carcinoma (10; 90.9%). The mean time after extended STL with THEP reconstruction was 34.36 months (±34.78 months) and median 15 months, ranging from 5 to 110 months. The mean time of nasogastric (NG) tube use was 43.18 days (±27.19 days) and tracheostomy 37 days (±27.68 days; median 26.5 days), and only one patient required permanent tracheostomy ( Table 1 ). All patients underwent neck dissection. No patient had a history of pneumonia.

**Table 1:** Demographic and clinical characteristics of patients submitted to extended supratracheal laryngectomies with tracheohyoidoepiglottopexy reconstruction

Patient	Age	Sex	Ethnicity	Years of study	Smoker upon diagnosis	Ethnic upon diagnosis	Clinical staging	Time after surgery (months)	NG tube (days)	TT (days)	RT	CT
1	59	Male	African descent	≤9	+	+	T3N0M0	5	26	25	-	-
2	58	Male	Caucasian	≤9	+	-	T3N0M0	15	20	26	-	-
3	68	Male	Caucasian	≤9	+	+	T3N0M0	15	92	75	-	-
4	73	Male	Caucasian	≤9	+	+	T3N0M0	13	31	27	+	-
5	69	Male	African descent	≤9	-	+	T3N0M0	12	40	fixed	-	-
6	72	Male	Caucasian	>9	+	+	T3N0M0	110	30	25	-	-
7	71	Male	Caucasian	>9	+	+	T3N0M0	16	27	27	-	-
8	71	Male	Caucasian	>9	-	-	T3N0M0	77	48	11	+	-
9	66	Male	Caucasian	>9	+	+	T3N0M0	33	33	26	-	-
10	83	Male	Caucasian	≤9	+	+	T3N0M0	13	28	28	-	-
11	52	Male	Caucasian	>9	+	+	T3N0M0	69	100	100	+	-

+ present; - absent.

NG: tube: nasogastric tube; TT: tracheostomy; RT: radiation therapy; CT: chemotherapy.

According to the scores obtained on the FCM scale, all patients in this study were exclusively orally fed and hydrated; 63.6% in level 7; 9.1% in level 6; 18.2% in level 5 and 9.1% in level 4. Aspiration and residue are described in table 2 ; the choice of not subdividing and specifying the tested consistencies was based on didactic purposes, better visualization and understanding of the results. The functional findings of the imaging study showed that four patients were aspirating at the time of the evaluation and all presented residue in different areas, with a higher frequency on base of tongue (10; 90.9%), valleculae (9; 81.82%) and arytenoids (9; 81.82%). In all aspirating patients, it occurred with the thin liquid consistency.

**Table 2:** Functional and clinical results of swallowing of patients submitted to extended supratracheal laryngectomies with tracheohyoidoepiglottopexy reconstruction

			Stasis						
			
Patient	Arytenoid	FCM ASHA NOMS	Aspiration	Tongue base	Posterior pharyngeal wall	Valleculae	Arytenoids	Upper esophageal sphincter	Pyriform sinuses
1	1	7	+	-	-	+	+	-	+
2	1	6	-	+	-	+	+	-	+
3	1	4	+	+	-	+	+	+	+
4	1	7	+	+	+	+	+	+	+
5	1	7	+	+	-	+	+	+	+
6	2	7	-	+	-	+	-	-	-
7	1	7	-	+	+	+	+	+	+
8	1	5	-	+	+	-	+	-	-
9	2	7	-	+	-	+	+	-	+
10	2	5	-	+	-	-	-	-	+
11	1	7	-	+	-	+	+	-	-
n (%)	-	-	4 (36.37)	10 (90.90)	3 (27.27)	9 (81.82)	9 (81.82)	4 (36.37)	8 (72.73)

+ present; - absent.

FCM/ASHA NOMS: Functional Communication Measures of Swallowing/American Speech-Language-Hearing Association National Outcome Measurement System (NOMS).

Data related to the evaluation of QoL in swallowing ( Table 3 ), demonstrated means above 80 for all scores (83.47 mean scores). Despite variations according to the aspects present in the protocol, the Global QoL was, for the majority, above 100 in 72.72% (n=8) of the patients.

**Table 3:** Impact of oncological treatment on quality of life in swallowing of patients submitted to extended supratracheal laryngectomy with tracheohyoidoepiglottopexy reconstruction

Patient	MDADI				

	Emotional	Functional	Physical	Global	Total
1	86.66	84	65	40	78.56
2	40	32	77.5	80	49.82
3	86.66	56	50	20	57.56
4	90	84	92.5	100	88.82
5	96.66	84	82.5	100	91.72
6	86.66	100	90	100	92.22
7	86.66	100	85	100	90.56
8	86.66	80	70	100	81.56
9	86.66	100	100	100	95.56
10	86.66	96	90	100	90.88
11	96.66	100	90	100	95.56
Average	84.54	83.27	81.14	85.45	82.98
SD	15.29	21.6	14.5	28.41	15.49

MDADI: M. D. Anderson Dysphagia Inventory; SD: standard deviation.

The results presented in table 4 were obtained through the evaluation of vocal quality and its effects on patients’ QoL. The overall severity and the presence of roughness were the highest means present in CAPE V (37.81 and 49.36, respectively). Regarding the QoL in voice, the physical and organic questions presented the highest averages, however, with effects considered mild in relation to the 40 questions in each area. The emotional range presented a minimum of zero and a maximum of 18 in its score, with an average of 6.36 (±7.05), and it was shown to be a minimally altered aspect. Regarding the total score of individuals, the mean of 33.36 (±22.56) had little impact on QoL from the perspective of vocal aspects.

**Table 4:** Perceptual-auditory analysis and quality of life in voice of patients submitted to extended supratracheal laryngectomy with tracheohyoidoepiglottopexy reconstruction

Patient	CAPE V						VHI			
	
	Overall severity	Roughness	Breathiness	Strain	Pitch	Loudness	Physical	Emotional	Organic	Total
1	42	50	1	1	5	5	15	10	12	37
2	39	35	0	0	8	8	30	18	30	78
3	60	70	5	5	10	30	12	12	26	50
4	50	65	46	40	42	42	28	18	18	64
5	32	50	5	5	5	0	7	4	19	30
6	34	64	37	30	10	10	7	0	6	13
7	50	50	9	9	9	35	15	1	7	23
8	36	29	0	0	9	18	11	1	17	29
9	16	15	0	0	5	5	0	0	5	5
10	33	56	0	0	7	7	6	0	6	12
11	24	59	9	9	15	9	16	6	4	26
Mean	37.81	49.36	10.18	9	11.36	15.36	13.36	6.36	13.63	33.36
SD	12.44	16.8	15.99	13.5	10.57	14	9.08	7.07	9	22.55

CAPE V: Consensus Auditory-Perceptual Evaluation of Voice; VHI: Voice handicap Index; SD: stadard deviation.

## DISCUSSION

Functional aspects of speech, deglutition and subsequent impacts on QoL were evaluated in 11 male patients over 50 years of age, agreeing with the findings of the only study to date on STL, in which 100% of 22 patients were male and 19 (86.36%) were aged ≥50 years.^( 4 , 19 , 23 )^ Both results are consistent with the population-based studies on head and neck cancer, which reported its highest occurrence in men with a mean age of approximately 60 years.^( 3 , 9 , 11 , 14 , 16 )^ Concomitant alcohol consumption and smoking was observed in more than 80% of patients in this study. Its synergistic effect is well-described in the literature, and these risk factors are associated with increased occurrence of head and neck cancer.^( 5 , 9 , 16 )^

More than half of the patients (n=6) had minimum education levels of elementary school (<9 years), an aspect of extreme importance, and one which agrees with studies that affirmed there is a greater prevalence of head and neck cancer in socioeconomically disadvantaged populations.^( 4 )^ Although the treatment of the patients in this study was carried out in a reference oncology hospital, which is part of the Brazilian public health system, the Unified Heath System (SUS - *Sistema Único de Saúde* ), being a public institution that receives predominantly patients with low income and schooling, therefore patients who may have little knowledge about the disease, its signs, symptoms and risk factors. This may represent a biased sample.

All individuals had T3 classification, with infiltrative and ulcerative tumors, which are in agreement with findings in the literature, stating that STL is traditionally prescribed for the treatment of tumors of intermediate and advanced stages.^( 2 , 14 , 15 )^ Considering the important anatomic resection of the surgical procedure, the restoration of swallowing was achieved after STL in the studied group, as well as in a similar group of 22 patients in Italy.^( 23 )^

The main objective of a partial intervention in the larynx is to obtain locoregional control of the disease and to enable laryngeal functions.^( 14 )^ According to the FCM scale, at the time of the evaluation, only one patient required moderate dietary modifications (Level 4), *i.e* . oral feeding management, through adaptations or consistency limitations. Even with the surgical extension larger than predicted in the original technique, the extended STL with THEP reconstruction in the assessed group proved to be a viable alternative to TL.^( 3 , 4 , 19 , 21 )^

In the literature, there was no consensus regarding the functional impact and the presence of one or two arytenoids in horizontal partial laryngectomy. However, when both CAU are preserved, according to Atallah et al.,^( 4 )^ and Leone et al.,^( 19 )^ the patient’s postoperative recovery occurs in a shorter time, and swallowing is more effective. Although our sample was too small to draw reliable conclusions on this, it is worthwhile to note that all individuals who maintained two CAU in their neolarynx presented less residue in the anatomical structures studied, absence of aspiration in the videofluoroscopic examination, without volume and consistency restrictions.

The most frequent functional complications in STL are usually related to swallowing.^( 9 , 11 , 13 , 16 , 20 - 22 )^ Thus, the presence of residue and aspiration in the VFSS analysis of patients in the present study was already partially predicted, as reported by some authors. The aspiration of the evaluated patients was mainly observed with the thin liquid consistency in controlled volumes. In 2017, Kaneoka et al.,^( 21 )^ reported the aspiration of water in a limited quantity is considered relatively benign, and is easily absorbed by the lungs, which would represent a minimal risk of aspiration pneumonia. Even if management of swallowing is not completely successful, it is possible that the amount of water aspirated be not sufficient to trigger pulmonary complications.^( 5 )^

In addition, the provision of thin liquids allows patients to appreciate the texture and taste of their preferred beverages, which probably promotes greater fluid intake and satisfaction with their diets.^( 5 )^ The benefits of providing thin liquids and exclusive oral feeding are improvement in the QoL.^( 5 , 17 )^

There was a greater impact on voice than on swallowing function, probably due to the resection of both vocal folds and vestibular folds.^( 3 , 23 )^ The overall severity and roughness were considered moderate characteristics in CAPE V, due to the structural and functional remnants of the resection. It is evident in the present study that roughness was the most prevalent aspect, being the most important negative functional trait in the voice of patients undergoing STL. The functional findings of the voice in the group were consistent with a similar study on STL conducted by Schindler et al.,^( 23 )^ in 2014, with evidence of moderate to severe roughness components and present report of mild to severe.

In the VHI, the patients presented lower mean values in the emotional field (6.36±7.07), and higher in the physical (13.36±9.08) and organic (13.63±9) fields, probably due to difficulties functional changes resulting from the surgical procedure, which, although modifying the physiology of vocal production, maintains the laryngeal voice, allowing for effective communication.

The voice of patients undergoing STL is produced through the passage of air through the neolarynx during expiration and consequent movement of the mucosa and remaining structures, such as arytenoids. In the literature, there is controversy over the impact of arytenoidectomy and the functional conditions of the remaining arytenoids on open horizontal partial laryngectomies.^( 18 )^ Buzaneli et al.,^( 17 )^ used CAPE V in a study with 12 patients undergoing SCL and found moderate dysphonia in participants who did not undergo arytenoidectomy, and severe dysphonia in patients with a single preserved CAU.^( 18 )^ These findings were also observed in the present study, in which the most serious results regarding the functionality and QoL in voice occurred in patients with only one CAU.^( 23 )^

All aspirating patients in this study had been submitted to resection less than 15 months ago, and presented stasis in several anatomical regions. Patients with over 15 months (n=5) of surgical resection did not aspirate and had less residue in the analyzed regions. Therefore, better functional and QoL results were found in patients with more than 1 year of STL in the evaluated group, suggesting the efficacy of long-term speech-language management and functional adaptation of the remaining structures.^( 3 , 12 , 23 )^

It is already known that in horizontal partial laryngectomies, such as STL and SCL, the patient must be willing to commit to extensive rehabilitation to maximize the potential for improvement.^( 9 , 19 )^ The successful management of patients in the study, who retained exclusively oral nutrition and hydration, and functional laryngeal voice, was observed with constant speech therapy at all times of treatment.

The STL extended in THEP reconstruction is a highly complex procedure performed in carefully selected patients, with contraindications: severe chronic obstructive pulmonary disease, uncontrolled diabetes mellitus, psychiatric syndromes, personal motivations, neurological problems that impair the ability to expectorate and/or swallow, or severe cardiac disease. Therefore, patients undergoing this procedure are at a lower risk of presenting postoperative complications.^( 9 , 19 , 23 )^

It is also important to point out that the presence of aspiration and residue did not affect the general health conditions of the individuals until the time of the evaluation. They adapted to their neolarynx and the new process of swallowing and vocal production in both the short and long term. This reinforces the need for follow-up by a specialized multiprofessional team at all times of treatment.

Among the limitations of this study are the small sample size and the time from surgery to the speech-language pathology evaluation. The results reiterate the efficacy of this surgical modality when integrated with speech-language rehabilitation, since all evaluated patients showed no evidence of disease and signaled good QoL in both voice and swallowing.

## CONCLUSION

The long-term and short-term results after extended supratracheal laryngectomy in tracheohyoidoepiglottopexy reconstruction showed that swallowing was sufficiently restored, allowing oral nutrition and hydration with positive quality of life scores on swallowing. Voice was very rough, indicating greater severity in this aspect. Patients demonstrated a good perception of the limitations related to voice disorders, and quality of life results suggested that oral communication was not significantly limited nor were there severe impacts on their activities of daily life. The presence of two cricoarytenoid units presented better functional results and quality of life in voice and swallowing. The evaluation of functional and quality of life results showed better scores in patients 12 months or more after surgery. All issues found in the study justify the indication of speech therapy for these patients at all times of treatment.
