# Supplementary material for: Swallowing, voice and quality of life of patients submitted to extended supratracheal laryngectomy
Source: Einstein (Sao Paulo). 2020 May 13;18:eAO5390. doi: 10.31744/einstein_journal/2020AO5390 (PMC7233281; doi:10.31744/einstein_journal/2020AO5390)
Supplement: Translation

# Translation: Deglutição, voz e qualidade de vida de pacientes submetidos à laringectomia supratraqueal alargada

**Authors:** Guilherme Maia Zica, Andressa Silva de Freitas, Ana Catarina Alves e Silva, Fernando Luiz Dias, Izabella Costa Santos, Emilson Queiroz Freitas, Hilton Augusto Koch

**Affiliations:** 1 Brasil Instituto Nacional de Câncer, Rio de Janeiro, RJ, Brasil .; 2 Brasil Fundação Oswaldo Cruz, Rio de Janeiro, RJ, Brasil .

**Keywords:** Laringectomia, Resultado do tratamento, Deglutição, Voz, Qualidade de vida

## Abstract

**Objetivo:**

Descrever os resultados funcionais e de qualidade de vida após a laringectomia supratraqueal alargada.

**Métodos:**

No período de setembro de 2009 a janeiro de 2018, 11 indivíduos do sexo masculino foram submetidos à laringectomia supratraqueal alargada. As habilidades de deglutição foram avaliadas por meio da videofluoroscopia e da escala clínica *Functional Communication Measures* . As vozes foram classificadas por análise perceptivo-auditiva da *Consensus Auditory-Perceptual Evaluation of Voice* . Todos os voluntários preencheram um questionário de autoavaliação para voz e deglutição.

**Resultados:**

A aspiração foi encontrada em quatro pacientes, e todos apresentaram estase em diferentes estruturas. Todos os sujeitos deste estudo apresentavam alimentação e hidratação exclusivas por via oral. Na avaliação da qualidade de vida na deglutição, os pacientes demonstraram médias >80 em todas as áreas (83,47 média dos escores). O grau geral e a presença de rugosidade foram os maiores escores médios na avaliação perceptivo-auditiva da voz (37,81 e 49,36 consecutivamente). A média de 33,36 (±22,56) demonstrou pouco impacto na qualidade de vida sob a perspectiva dos aspectos vocais.

**Conclusão:**

Após a laringectomia supratraqueal, a deglutição foi suficientemente restaurada, e a qualidade de vida foi satisfatória. A voz apresenta qualidade gravemente comprometida com comunicação oral preservada, demonstrando baixo impacto nas atividades da vida diária. Todos os indivíduos que mantiveram duas unidades cricoaritenóideas apresentaram melhores resultados funcionais na deglutição e na voz.

## INTRODUÇÃO

O câncer de laringe é a segunda doença maligna respiratória mais comum no mundo, sendo um dos locais de maior incidência dos tumores de cabeça e pescoço. Apresenta-se majoritariamente em homens acima dos 40 anos de idade, e seu tipo histológico mais prevalente é o carcinoma epidermoide, confirmado em mais de 90% dos casos.^( 1 , 2 )^O prognóstico é insatisfatório e limitado quando a doença é diagnosticada em estágios avançados, prejudicando a qualidade de vida (QV) do indivíduo, principalmente considerando alterações de voz e deglutição.^( 1 , 3 - 6 )^

O tratamento do câncer de laringe por meio de laringectomia total (LT), com ou sem radioterapia (RT), altera significativamente a QV dos pacientes. A retirada completa do órgão gera grandes impactos oriundos das alterações nas capacidades de fonar e deglutir.^( 7 , 8 )^Há estudos que mostram que a RT e a quimioterapia (QT) são alternativas à laringectomia primária, evitando a desfiguração, uma traqueostomia permanente e complicações, como fístula faringocutânea, infecções e ruptura da artéria carótida.^( 6 - 9 )^No entanto, existem evidências, principalmente em estágios avançados, de que as sequelas dessas técnicas também podem ser drásticas, resultando na preservação de um órgão não funcional com alterações estruturais significativas.^( 8 , 10 )^

Diante das falhas do tratamento com RT e da baixa eficácia dos protocolos não cirúrgicos com altos níveis de toxicidade, as laringectomias parciais horizontais abertas (LPH) ressurgem em centros especializados como alternativa viável aos demais procedimentos.^( 10 )^Essa técnica cirúrgica conservadora para câncer de laringe remove o tumor e sua margem de segurança livre da doença, reconstruindo uma neolaringe funcional.^( 3 , 10 , 11 )^

As laringectomias parciais horizontais se consagraram como opção cirúrgica para o tratamento do câncer de laringe nos estágios intermediário/avançado.^( 12 )^Em 1959, a laringectomia supracricóidea (LSC) com cricohioidoepiglotopexia foi realizada por Majer, como ressecção oncológica segura para pacientes com câncer de laringe.^( 13 )^Como método atual e análogo, surgiu a laringectomia supratraqueal (LST), para o tratamento de tumores laríngeos com extensão subglótica e invasão anterior da cartilagem cricóidea.^( 3 , 14 , 15 )^

A técnica cirúrgica da LST consiste na ressecção da cartilagem tireóidea e seu espaço paraglótico; na preservação da parte posterior da cartilagem cricóide; e na manutenção do osso hioide, da epiglote e de pelo menos uma cartilagem aritenoide.^( 12 , 13 , 16 )^Sua reconstrução é descrita com duas variações: traqueohioidopexia, que consiste na manutenção de ambas ou de uma única unidade cricoaritenoide (UCA); traqueohioidoepiglotopexia (THEP) com preservação da epiglote, mantendo duas ou uma única UCA.^( 17 , 18 )^Nos casos em que a ressecção é mais ampla do que o previsto pela técnica descrita, como nos relatos de LST com extensão à base da língua, ao tecido adjacente ou às aritenoides, acrescentamos à nomenclatura o termo LST “ampliada” ou “modificada”.^( 19 - 21 )^

Na literatura especializada, há evidências de complicações funcionais após LPH em relação à respiração, à voz e à deglutição, com baixa incidência de traqueostomias permanentes.^( 9 , 22 )^No entanto, a correlação dos efeitos funcionais na QV dos pacientes submetidos à LST, até o momento, só foi sugerida em um único estudo, realizado em 2015.^( 23 )^Seus achados preliminares descreveram a efetividade da técnica em um grupo de 22 pacientes, na Itália, com manutenção da alimentação oral exclusiva em 20 pacientes e melhores resultados de QV e voz, em comparação aos escores de deglutição devido às mudanças na dieta.^( 23 )^Assim, é de suma importância que a população de países em desenvolvimento também seja caracterizada sob esses aspectos, em suas reais condições e contextos socioeconômicos e demográficos.

## OBJETIVO

Avaliar e descrever os resultados funcionais e de qualidade de vida de deglutição e voz em pacientes submetidos à laringectomia supratraqueal alargada com traqueohioidoepiglotopexia em um hospital oncológico de referência da América Latina.

## MÉTODOS

Estudo transversal observacional, aprovado pelo Comitê de Ética em Pesquisa da instituição sob CAAE: 26331314.2.0000.5274, parecer 616.249. Foram incluídos indivíduos de ambos os sexos, matriculados no período de 2009 a 2018, no Setor de Cabeça e Pescoço do Hospital do Câncer I do Instituto Nacional de Câncer, na cidade do Rio de Janeiro (RJ) e submetidos à LST com reconstrução alargada da THEP. Os critérios de exclusão foram pacientes com menos de 18 anos de idade, não cooperantes devido a comprometimentos linguístico-cognitivos e submetidos a outro tipo de intervenção cirúrgica na região laríngea após LST com THEP alargada. Todos os voluntários participantes do estudo assinaram o Termo de Consentimento Livre e Esclarecido.

Os indivíduos elegíveis para o estudo foram localizados no sistema cirúrgico de acordo com o corte temporal preestabelecido. Foram coletadas informações demográficas e clínicas por meio da análise de prontuários físicos e eletrônicos. Para as avaliações fonoaudiológicas e de QV, os pacientes foram agendados e participaram dos procedimentos descritos a seguir.

### Avaliação funcional e qualidade de vida em deglutição

Foram utilizados estudos videofluoroscópicos da deglutição (VFD) para avaliar a deglutição dos pacientes do estudo. Os exames foram realizados utilizando o equipamento Siemens AXIOM *Iconos Remote Control* X-ray (número de série 13020) e avaliados por meio de um protocolo de avaliação videofluoroscópica da deglutição, com base em Logemann.^( 24 )^Todos os segmentos de vídeo foram gravados no plano de visão lateral, com taxa de captura de 30 quadros por segundo.

O preparo das consistências foi feito da seguinte maneira: o contraste foi oferecido em um copo, com manuseio livre e usando diluição de 100% de sulfato de bário (SB) Bariogel^®^, água mineral e o espessante Resource^®^ThickenUp Clear. Avaliamos os pacientes com três consistências: consistência líquida em 5mL (2,5mL de água + 2,5mL de SB), 10mL (5mL de água + 5mL de SB) e 20mL (10mL de água + 10mL de SB); consistência semilíquida em 5mL de SB, 10mL de SB e 20mL de SB; e consistência pastosa em 5mL (5mL de SB + 1,2g de espessante), 10mL (10mL de SB + 2,4g de espessante) e 20mL (20mL de SB + 3,6g de espessante). Para padronizar o exame, não foi incluída a consistência sólida, devido à existência de indivíduos com ausências dentárias importantes. Foi utilizada a Escala de Penetração-Aspiração (PAS - The *Penetration-Aspiration Scale* ) desenvolvida por Rosenbek et al., para analisar o exame fluoroscópico.^( 25 )^

A *Functional Communication Measures of Swallowing* (FCM) da *American Speech-Language-Hearing Association* (ASHA) *National Outcome Measurement System* (NOMS) foi utilizada como meio subjetivo e complementar na análise da ingestão oral. A escala se baseia na observação clínica, sendo amplamente aceita na avaliação da disfagia orofaríngea, com escores que variam de 1 (menos funcional) a 7 (normal).^( 26 )^

A única ferramenta disponível para avaliar os efeitos das alterações da deglutição na QV em indivíduos em tratamento para câncer de cabeça e pescoço é o Questionário de Disfagia M. D. Anderson (MDADI - *M. D. Anderson Dysfaghia Inventory* ), que foi desenvolvido e validado por Chen et al.,^( 27 )^composto por 20 questões, sendo uma global e as demais segmentadas em domínios emocional, funcional e físico.^( 28 , 29 )^Todas as escalas apresentam cinco possibilidades de resposta, variando de 1 (concordo totalmente) a 5 (discordo totalmente), exceto um item da subescala emocional (E7) e um item da subescala funcional (F2), que pontuam-se variando de 5 (concordo totalmente) a 1 (discordo totalmente). O escore de cada subescala é obtido a partir do cálculo da média da pontuação de seus itens, multiplicados por 20. O escore total é obtido a partir da média dos escores de cada domínio (emocional, funcional e físico) multiplicado por 20. O indivíduo pode obter uma pontuação variando de zero a cem, e quanto menor o valor do escore observado, pior o efeito da disfagia em sua QV e funcionalidade.^( 27 , 28 )^

### Avaliação perceptiva e qualidade de vida referentes à voz

Os pacientes deste estudo foram avaliados quanto à qualidade vocal usando um método de análise perceptivo-auditiva desenvolvido em 2009 por Kempster et al.,^( 29 )^a classificação *Consensus Auditory-Perceptual Evaluation of Voice* (CAPE V). O objetivo dessa escala é qualificar e quantificar o sinal sonoro, permitindo determinar a qualidade vocal do indivíduo no contexto do estudo da voz. A análise do ouvinte é passada para uma escala linear de 100mm, com pontuações determinadas pela localização da marca escolhida (variando de zero a cem) por um fonoaudiólogo previamente treinado. Os aspectos avaliados são representados pelo grau geral do desvio vocal do paciente, presença e grau de aspereza, soprosidade e tensão, e variações de *pitch* e *loudness* . Quanto mais próximo de cem for o valor do escore obtido, pior é a qualidade vocal do paciente.^( 29 )^

Para investigar o impacto das alterações vocais na QV do paciente, foi aplicado o questionário Índice de Desvantagem Vocal (IDV), que foi traduzido e adaptado para o português brasileiro em 2009 por Behlau et al.,^( 30 )^a partir do *Voice Handicap Index* (VHI), e permite interpretar a percepção do paciente sobre a disfonia e as repercussões em sua QV. O protocolo é composto por 30 itens que avaliam 3 domínios − funcional (10), orgânico (10) e emocional (10) −, quantificados em uma escala Likert de 5 pontos (0-4), cuja pontuação é calculada por um somatório simples. Os resultados obtidos podem variar de zero a 120, e quanto maior for o escore, mais importante o comprometimento da QV do paciente.^( 30 )^

## RESULTADOS

Foram avaliados 11 pacientes, com média de idade de 67,45 anos (±8,5 anos) e mediana de 69 anos, 9 autodeclarados brancos (81,8%) e 2 negros (18,2%) ( Tabela 1 ). Do grupo estudado, 45,5% confirmaram história familiar de câncer. A maioria dos indivíduos apresentava diagnóstico histopatológico de carcinoma de células escamosas (10; 90,9%). O tempo médio após LST alargada com reconstrução da THEP foi de 34,36 meses (±34,78 meses) e mediana de 15 meses, variando de 5 a 110 meses. O tempo médio de uso de sonda nasogástrica (SNG) foi de 43,18 dias (±27,19 dias) e de traqueostomia 37 dias (±27,68 dias; mediana 26,5 dias); apenas um paciente necessitou de traqueostomia permanente ( Tabela 1 ). Todos os pacientes foram submetidos à linfadenectomia cervical. Nenhum paciente teve histórico de pneumonia.

**Tabela 1:** Características demográficas e clínicas dos pacientes submetidos à laringectomia supratraqueal alargada com reconstrução por meio da traqueohioidoepiglotopexia

Paciente	Idade	Sexo	Etnia	Escolaridade	Tabagista ao diagnóstico	Etilista ao diagnóstico	Estadiamento clínico	Tempo pós-cirurgia (meses)	SNG (dias)	TQT (dias)	RT	QT
1	59	Masculino	Negro	≤9	+	+	T3N0M0	5	26	25	-	-
2	58	Masculino	Branco	≤9	+	-	T3N0M0	15	20	26	-	-
3	68	Masculino	Branco	≤9	+	+	T3N0M0	15	92	75	-	-
4	73	Masculino	Branco	≤9	+	+	T3N0M0	13	31	27	+	-
5	69	Masculino	Negro	≤9	-	+	T3N0M0	12	40	fixo	-	-
6	72	Masculino	Branco	>9	+	+	T3N0M0	110	30	25	-	-
7	71	Masculino	Branco	>9	+	+	T3N0M0	16	27	27	-	-
8	71	Masculino	Branco	>9	-	-	T3N0M0	77	48	11	+	-
9	66	Masculino	Branco	>9	+	+	T3N0M0	33	33	26	-	-
10	83	Masculino	Branco	≤9	+	+	T3N0M0	13	28	28	-	-
11	52	Masculino	Branco	>9	+	+	T3N0M0	69	100	100	+	-

+ presente; - ausente.

SNG: tubo nasogástrico; TQT: traqueostomia; RT: radioterapia; QT: quimioterapia.

De acordo com os escores obtidos na escala FCM, todos os pacientes deste estudo estavam com alimentação e hidratação exclusivas por via oral: 63,6% no nível 7; 9,1% no nível 6; 18,2% no nível 5; e 9,1% no nível 4. A aspiração e o resíduo foram organizados na tabela 2 . A escolha de não subdividir e especificar as consistências testadas foi pautada na didática, melhor visualização e compreensão dos resultados. Os achados funcionais do estudo da deglutição por imagem mostraram que quatro pacientes apresentavam aspiração no momento da avaliação, e todos possuíam resíduo em diferentes áreas, com maior frequência na base da língua (10; 90,9%), valéculas (9; 81,82%) e aritenoides (9; 81,82%). Em todos os pacientes aspirativos, o episódio ocorreu com a consistência líquida fina.

**Tabela 2:** Resultados funcionais e clínicos da deglutição de pacientes submetidos à laringectomia supratraqueal alargada com reconstrução por meio da traqueohioidoepiglotopexia

			Resíduo						
			
Paciente	Ariteoide	FCM ASHA NOMS	Aspiração	Base da língua	Parede posterior da faringe	Valécula	Aritenoides	Esfíncter esofágico superior	Recessos piriformes
1	1	7	+	-	-	+	+	-	+
2	1	6	-	+	-	+	+	-	+
3	1	4	+	+	-	+	+	+	+
4	1	7	+	+	+	+	+	+	+
5	1	7	+	+	-	+	+	+	+
6	2	7	-	+	-	+	-	-	-
7	1	7	-	+	+	+	+	+	+
8	1	5	-	+	+	-	+	-	-
9	2	7	-	+	-	+	+	-	+
10	2	5	-	+	-	-	-	-	+
11	1	7	-	+	-	+	+	-	-
n (%)	-	-	4 (36,37)	10 (90,90)	3 (27,27)	9 (81,82)	9 (81,82)	4 (36,37)	8 (72,73)

+ presente; - ausente.

FCM/ASHA NOMS: *Functional Communication Measures of Swallowing/American Speech-Language-Hearing Association National Outcome Measurement System* (NOMS).

Os dados da avaliação da QV referente à deglutição ( Tabela 3 ) mostraram médias acima de 80 para todos os escores (83,47 escores médios). Apesar das variações em relação aos aspectos presentes no protocolo, a QV global ficou, para a maioria, acima de 100 em 72,72% (n=8) dos pacientes.

**Tabela 3:** Impacto do tratamento oncológico na qualidade de vida referente à deglutição em pacientes submetidos à laringectomia supratraqueal alargada com reconstrução traqueohioidoepiglotopexia

Paciente	MDADI				

	Emocional	Funcional	Físico	Global	Total
1	86,66	84	65	40	78,56
2	40	32	77,5	80	49,82
3	86,66	56	50	20	57,56
4	90	84	92,5	100	88,82
5	96,66	84	82,5	100	91,72
6	86,66	100	90	100	92,22
7	86,66	100	85	100	90,56
8	86,66	80	70	100	81,56
9	86,66	100	100	100	95,56
10	86,66	96	90	100	90,88
11	96,66	100	90	100	95,56
Média	84,54	83,27	81,14	85,45	82,98
DP	15,29	21,6	14,5	28,41	15,49

MDADI: Questionário de Disfagia M. D. Anderson; DP: desvio padrão.

Os resultados da tabela 4 foram obtidos por meio da avaliação da qualidade vocal e suas repercussões na QV dos pacientes. O grau geral e a presença de rugosidade foram as maiores médias presentes no CAPE V (37,81 e 49,36, respectivamente). Referente à QV em voz, os aspectos orgânico e físico apresentaram as maiores médias, no entanto, com afecção considera leve em relação às 40 questões de cada área. O âmbito emocional apresentou mínimo de zero e máxima de 18 em sua pontuação, com média de 6,36 (±7,05), e demonstrou ser um aspecto pouco alterado. Com relação ao escore total dos indivíduos, a média de 33,36 (±22,56) representou pouco impacto na QV sob perspectiva dos aspectos vocais.

**Tabela 4:** Análise perceptivo-auditiva e de qualidade de vida referente à voz dos pacientes submetidos à laringectomia supratraqueal alargada com reconstrução traqueohioidoepiglotopexia

Paciente	CAPE V						IDV			
	
	Grau geral	Rugosidade	Soprosidade	Tensão	*Pitch*	*Loudness*	Físico	Emocional	Orgânico	Total
1	42	50	1	1	5	5	15	10	12	37
2	39	35	0	0	8	8	30	18	30	78
3	60	70	5	5	10	30	12	12	26	50
4	50	65	46	40	42	42	28	18	18	64
5	32	50	5	5	5	0	7	4	19	30
6	34	64	37	30	10	10	7	0	6	13
7	50	50	9	9	9	35	15	1	7	23
8	36	29	0	0	9	18	11	1	17	29
9	16	15	0	0	5	5	0	0	5	5
10	33	56	0	0	7	7	6	0	6	12
11	24	59	9	9	15	9	16	6	4	26
Média	37,81	49,36	10,18	9	11,36	15,36	13,36	6,36	13,63	33,36
DP	12,44	16,8	15,99	13,5	10,57	14	9,08	7,07	9	22,55

CAPE V: *Consensus Auditory-Perceptual Evaluation of Voice* ; IDV: Índice de Desvantagem Vocal; DP: desvio padrão.

## DISCUSSÃO

Foram avaliados os aspectos funcionais da fala, da deglutição e os subsequentes impactos na QV em 11 pacientes do sexo masculino com mais de 50 anos de idade, concordando com os achados do único estudo realizado até o momento sobre LST, no qual 100% dos 22 pacientes eram do sexo masculino e 19 (86,36%) tinham 50 anos ou mais.^( 4 , 19 , 23 )^Ambos os resultados são consistentes com os estudos populacionais do câncer de cabeça e pescoço, que relataram sua maior ocorrência em homens com média de idade de aproximadamente 60 anos.^( 3 , 9 , 11 , 14 , 16 )^Foi observado consumo concomitante de álcool e tabaco em mais de 80% dos pacientes deste estudo. Seu efeito sinérgico é bem descrito na literatura, associado como fatores de risco para ocorrência de câncer de cabeça e pescoço.^( 5 , 9 , 16 )^

Mais da metade dos pacientes (n=6) apresentava escolaridade até o Ensino Fundamental (<9 anos), um aspecto de extrema importância e que concorda com estudos que afirmaram haver maior prevalência de câncer de cabeça e pescoço em populações de nível socioeconômico desfavorecido.^( 4 )^Embora o tratamento dos pacientes deste estudo tenha sido realizado em hospital de referência em oncologia, este faz parte do sistema de saúde público brasileiro, o Sistema Único de Saúde (SUS), sendo uma instituição pública que recebe predominantemente pacientes de baixa renda e escolaridade, que são, portanto, indivíduos com pouco conhecimento sobre a doença, seus sinais, sintomas e fatores de risco. Isso pode representar uma amostra tendenciosa.

Todos os indivíduos tinham classificação T3, com tumores infiltrativos e ulcerativos, concordando com os achados da literatura, segundo os quais a LST é tradicionalmente indicada para o tratamento de tumores de estágio intermediário e avançado.^( 2 , 14 , 15 )^Levando em consideração a importante ressecção anatômica do procedimento cirúrgico, a restauração da deglutição foi alcançada após a LST no grupo estudado, bem como em um grupo semelhante de 22 pacientes na Itália.^( 23 )^

O principal objetivo de uma intervenção parcial na laringe é obter o controle locorregional da doença e possibilitar a manutenção das funções laríngeas.^( 14 )^De acordo com a escala FCM, no momento da avaliação, apenas um paciente necessitava de modificações moderadas na dieta (nível 4), ou seja, manejo da alimentação oral por meio de adaptações ou restrições de consistência. Mesmo com a extensão cirúrgica mais ampla do que o previsto na técnica original, a LST alargada com reconstrução por meio da THEP no grupo avaliado mostrou-se alternativa viável à LT.^( 3 , 4 , 19 , 21 )^

Na literatura, não houve consenso sobre o impacto funcional e a presença de uma ou duas aritenoides na LPH. No entanto, quando as duas UCA são preservadas, segundo Atallah et al.,^( 4 )^e Leone et al.,^( 19 )^a recuperação pós-operatória do paciente ocorre em menor tempo e a deglutição é mais eficaz. Embora nossa amostra seja pequena demais para tirar conclusões definitivas, vale ressaltar que todos os indivíduos que mantiveram duas UCAs em sua neolaringe apresentaram menos resíduo nas estruturas anatômicas avaliadas e ausência de aspiração no exame videofluoroscópico, sem restrições de volume e consistência.

As complicações funcionais mais frequentes na LST estão geralmente relacionadas à deglutição.^( 9 , 11 , 13 , 16 , 20 - 22 )^Assim, a presença de resíduo e aspiração na análise VFD dos pacientes do presente estudo já era parcialmente prevista, conforme relatado por alguns autores. Nos pacientes avaliados, observou-se aspiração principalmente com a consistência líquida fina em volumes controlados. Em 2017, Kaneoka et al.,^( 21 )^relataram que a aspiração de água em pequena quantidade é considerada relativamente benigna, sendo facilmente absorvida pelos pulmões, o que representaria risco mínimo de pneumonia por broncoaspiração. Mesmo que o controle da deglutição não seja completamente bem-sucedido, é possível que a quantidade de água aspirada não seja suficiente para desencadear complicações pulmonares.^( 5 )^

Além disso, o fornecimento de líquidos finos permite que os pacientes apreciem a textura e o sabor de suas bebidas preferidas, o que provavelmente promove maior ingestão de líquidos e maior satisfação oral com a dieta.^( 5 )^A oferta de líquidos finos e alimentação exclusivamente por via oral traz como benefício uma melhoria na QV.^( 5 , 17 )^É preciso equilíbrio entre a segurança, a eficácia e o conforto no processo de reabilitação da deglutição.

Houve maior impacto na voz do que na função da deglutição, provavelmente devido à ressecção tanto das pregas vocais quanto das pregas vestibulares.^( 3 , 23 )^O grau geral e a rugosidade foram consideradas características moderadas no CAPE V, devido aos resquícios estruturais e funcionais da ressecção. É evidente, no presente estudo, que a rugosidade foi o aspecto mais prevalente, sendo característica funcional negativa mais importante na voz dos pacientes submetidos a LST. Os achados funcionais referentes à voz no grupo foram consistentes com os de estudo semelhante sobre LST, realizado por Schindler et al.,^( 23 )^em 2014, que apresentava evidência de componentes de rugosidade de moderados a graves e soprosidade presente de leve a grave.

No IDV, os pacientes apresentaram valores médios mais baixos no campo emocional (6,36±7,07) e mais elevados nos campos físico (13,36±9,08) e orgânico (13,63±9), provavelmente devido às dificuldades funcionais decorrentes do procedimento cirúrgico, as quais, embora modifiquem a fisiologia da produção vocal, mantêm a voz da laringe, permitindo uma comunicação eficaz.

A voz dos pacientes submetidos à LST é produzida por meio da passagem do ar pela neolaringe durante a expiração e consequente movimentação da mucosa e estruturas remanescentes, como as aritenoides. Na literatura, em voz, há controvérsia sobre o impacto da aritenoidectomia e as condições funcionais das aritenoides remanescentes nas LPH abertas.^( 18 )^Buzaneli et al.,^( 17 )^utilizaram o CAPE V em estudo com 12 pacientes submetidos à LSC e encontraram disfonia moderada em participantes não submetidos à aritenoidectomia e disfonia grave nos pacientes com uma única UCA preservada.^( 18 )^Esses achados também foram observados no presente estudo. Os resultados mais graves referentes à funcionalidade e QV da voz ocorreram nos pacientes com uma única UCA.^( 23 )^

Todos os pacientes que apresentaram aspiração neste estudo tinham sido operados há menos de 15 meses e apresentavam estase em várias regiões anatômicas. Os pacientes com mais de 15 meses (n=5) de ressecção cirúrgica não aspiraram e apresentaram menos resíduo nas regiões analisadas. Portanto, foram encontrados melhores resultados funcionais e de QV em pacientes com mais de 1 ano de LST no grupo, sugerindo a eficácia do tratamento fonoaudiológico a longo prazo e a adaptação funcional das estruturas remanescentes.^( 3 , 12 , 23 )^

Sabe-se que, nas LPH, como a LST e a LSC, o paciente precisa estar disposto a se comprometer a uma ampla reabilitação, para maximizar o potencial de melhora.^( 9 , 19 )^O sucesso terapêutico dos pacientes do estudo, que apresentam nutrição e hidratação exclusivas por via oral e voz laríngea funcional, foi possível por meio do atendimento fonoaudiológico constante em todos os momentos do tratamento.

A LST alargada com reconstrução da THEP é um procedimento altamente complexo, realizado em pacientes cuidadosamente selecionados, tendo as seguintes contraindicações: doença pulmonar obstrutiva crônica grave, *diabetes mellitus* não controlado, síndromes psiquiátricas, motivações pessoais, problemas neurológicos que prejudicam a capacidade de expectorar e/ou deglutir e doença cardíaca grave. Portanto, os pacientes submetidos a esse procedimento apresentam menor risco de complicações pós-operatórias.^( 9 , 19 , 23 )^

É importante ressaltar que a presença de aspiração e resíduo não afetaram as condições gerais de saúde dos indivíduos até o momento da avaliação. Eles se adaptaram à sua neolaringe e ao novo processo de deglutição e produção vocal a curto e longo prazo. Isso reforça a necessidade de acompanhamento por uma equipe multiprofissional especializada em todos os momentos do tratamento.

Entre as limitações deste estudo estão o pequeno tamanho da amostra e o tempo entre a cirurgia e a avaliação fonoaudiológica. Os resultados reiteram a eficácia dessa modalidade cirúrgica quando integrada à reabilitação fonoaudiológica, uma vez que todos os pacientes avaliados não apresentaram evidências de doença e sinalizaram boa QV na voz e na deglutição.

## CONCLUSÃO

Os resultados a longo e a curto prazo pós-laringectomia supratraqueal alargada com reconstrução por meio de traqueohioidoepiglotopexia mostraram que a deglutição foi suficientemente restaurada, permitindo nutrição e hidratação orais com escores positivos de qualidade de vida na deglutição. A voz era muito rugosa/áspera e soprosa, indicando maior gravidade nesse aspecto. Os pacientes demonstraram boa percepção das limitações relacionadas aos distúrbios da voz, e os resultados da avaliação de qualidade de vida sugeriram que a comunicação oral não foi significativamente limitada, nem houve graves impactos em suas atividades cotidianas. A presença de duas unidades cricoaritenóideas apresentou melhores resultados funcionais e de qualidade de vida na voz e na deglutição. A avaliação dos resultados funcionais e de qualidade de vida mostrou melhores pontuações nos pacientes com 12 meses ou mais de pós-operatório. Todas as queixas e disfunções observadas no estudo justificam a indicação de terapia fonoaudiológica para esses pacientes em todos os momentos do tratamento.
